# Cigarette Smoke and Cancer

**DOI:** 10.1155/2011/172678

**Published:** 2011-08-29

**Authors:** Sushant Kachhap, Venkateshwar G. Keshamouni, David Z. Qian, Aditi Chatterjee

**Affiliations:** ^1^Department of Otolaryngology-Head and Neck Surgery, Head and Neck Cancer Research Division, Johns Hopkins University School of Medicine, Baltimore, MD 21205-2196, USA; ^2^Division of Pulmonary and Critical Care Medicine, Department of Internal Medicine, University of Michigan Medical Center, Ann Arbor, MI 48109, USA; ^3^Prostate Cancer/Genitourologic Program, The Sidney Kimmel Cancer Center, Johns Hopkins Medical Institute, Baltimore, MD 21205-2196, USA; ^4^Graduate Faculty in Cancer Biology, OHSU Knight Cancer Institute, Oregon Health and Science University, Portland, OR 97239-3098, USA

Increased tobacco smoke exposure positively correlates with a wide variety of cancers including those of lung, breast, pancreas, and prostate cancer. There are about 250 chemicals present in tobacco that have been linked to cancer. Smokeless tobacco, in the form of chewed tobacco leaves, snuff, and betel quid, has also been linked to oral and pancreatic cancers. Recent efforts to decipher mechanisms by which tobacco-derived carcinogens induce various cancers have provided profound insights about signaling pathways that are perturbed by these compounds, leading to oncogenic signaling. This special issue collates reviews and research articles that provide insights about tobacco-induced cancers at molecular, clinical, and epidemiological level.

Emphasizing the use of tobacco as a global health concern, Oppeltz et al. provide a detailed review highlighting the trend towards increased tobacco use and the increasing cancer burden in developing countries. Developing countries, such as Taiwan, may indeed be at risk which is underscored by the study of Lin et al., who evaluated a prospective study cohort and found that habitual cigarette smokers, alcohol consumers, and betel quid chewers have a higher risk of contracting oral cancer. This study finds an alarming 40-fold risk of developing oral cancer in individuals who have all the above habits than controls. 

The risk of cancer is not limited to smokers but also affects individuals who are indirectly exposed to tobacco-derived carcinogens. However, the link between paternal smoking and childhood leukemia is not yet clearly established. Using meta-analysis, an interesting review article by Liu et al. draws a link between paternal smoking and childhood acute lymphoblastic leukemia. Pancreatic cancers have been linked to tobacco use. A research article by Lochan et al. further implicates family history as an important factor promoting cancers among smokers. The authors find that individuals with a family history of malignancy are at an increased risk of pancreatic cancer. Furthermore, individuals with a family history of malignancy and who smoke appear to require a lesser degree of tobacco exposure for the development of pancreatic cancer. To curb the use of tobacco, it is necessary to provide professional smoking cessation aid. However, smoking cessation intervention should be tailored depending on the population and ethnicity, and behavioral and cultural differences should be taken into account. A study by Delnevo et al. indicates the heterogeneous nature of tobacco use among South Asian immigrants in the USA. The study drives an important point of segregating tobacco users depending on their country of origin, and not just grouping them as “Asians,” for a more reliable understanding into the behavior of tobacco use in this population. A short clinical report by Mazza et al. emphasizes the need for smoking cessation clinics in comprehensive cancer centres to benefit smoker cancer patients. 

Understanding tobacco-induced cancers at the molecular level is key for developing biomarkers and therapeutics for early intervention. Chen et al. provide a comprehensive review about epigenetic and molecular mechanisms that are deregulated by tobacco carcinogens with special emphasis on nicotine, N-nitrosodiethylamine, 4-(methylnitrosamino)-1-(3-pyridyl)-1-butanone (NNK), and polyaromatic hydrocarbon. This paper highlights the complex nature of tobacco-induced carcinogenesis and provides recent updates on molecular targets which include receptors, cell cycle regulators, mitogen-activated protein kinases, apoptosis mediators, angiogenic factors, and invasion and metastasis mediators. A research article by Dev et al. explores the molecular mechanism of cigarette-smoke—induced proliferation of lung cells. They found that p-benzoquinone, in aqueous smoke extract, binds and modifies EGFR, preventing its degradation leading to increased EGFR signaling and proliferation. Chaudhry et al., on the other hand, report the effects of brief exposure to tobacco-derived carcinogens, including NNK, on cellular activity, morphology, and gene expression of bronchial epithelial cells. Knowledge gained from *in vitro* work has been extended to *in vivo* models. Recent advances in transgenic and knockout animal models have provided unprecedented opportunity to selectively perturb molecular pathways and understand its role in tobacco-induced carcinogenesis. Zheng et al. provide a comprehensive review of our current understanding of pathways altered by NNKs using such animal models. 

Search for reliable biomarkers for early detection of lung and oral cancer is an active area of research. Recent advances in tools to probe epigenetic changes in the DNA have included DNA methylation in the repertoire of biomarkers of predictive and prognostic importance. Using MethyLight assays, Salskov et al. investigated hypermethylation in lung tissues in a cohort of smokers and nonsmokers for nineteen gene promoters. Their data suggests hypermethylation of CCND2 could reflect smoking-induced precancerous changes in the lung. Although several compounds in tobacco are proven carcinogens, nicotine, the main addictive compound in tobacco, is not carcinogenic. Two review articles, one by Singh et al. and the other by Lee et al., describe the role of nicotine in carcinogenesis. While Singh et al. examine the historical data connecting nicotine tumor progression with updates on recent efforts to target the nicotinic acetylcholine receptors to combat cancer, Lee et al. provide recent updates on the assembly, activity, and biological functions of nicotinic receptors, with current understanding regarding developments in the therapeutic application of nicotinic receptor ligands. Carcinogens present in cigarette smoke are not always intrinsic to tobacco leaves; fertilizer-derived carcinogens and microbial toxins could also contribute to carcinogenesis. Review articles by Zaga et al. about radioactive carcinogens, Pb-210 and Po-210, which accumulate in tobacco leaves, and by Pauly et al. about microbes and microbial toxins in cigarettes, provide important insight into the interesting cancer-promoting milieu of tobacco smoke.

*Sushant Kachhap* *Sushant Kachhap* *Venkateshwar G. Keshamouni* *Venkateshwar G. Keshamouni* *David Z. Qian* *David Z. Qian* *Aditi Chatterjee* *Aditi Chatterjee*

